# Kinematic Assessment of the Physician’s Body Position and Musculoskeletal Loads During Breast and Abdominal Ultrasound Examinations

**DOI:** 10.3390/jcm14207417

**Published:** 2025-10-20

**Authors:** Mateusz Winder, Maria Hankus, Marcin Ciekalski, Izabela Rosół, Anna Miller-Banaś, Agata Guzik-Kopyto, Katarzyna Steinhof-Radwańska, Robert Michnik

**Affiliations:** 1 Department of Radiology and Nuclear Medicine, Medical University of Silesia, 40-055 Katowice, Poland; 2 Students Scientific Society, Department of Biomechatronics, Silesian University of Technology, 41-800 Zabrze, Poland; 3 Students Scientific Society, Department of Radiology and Nuclear Medicine, Medical University of Silesia, 40-055 Katowice, Poland; 4 Department of Biomechatronics, Silesian University of Technology, 41-800 Zabrze, Poland

**Keywords:** ultrasonography, kinematics, biomechanics, ergonomics, breast ultrasound, abdominal ultrasound

## Abstract

**Background**: Ultrasound is a non-invasive imaging technique that provides real-time evaluation of anatomical structures. While versatile in examining various organs, it can be physically demanding for physicians due to the need for challenging positions, causing musculoskeletal pain and potentially work-related diseases over time. The study aimed to assess the ergonomics of abdominal and breast ultrasound, identify the most challenging anatomical area, determine which part of the examination causes the greatest strain, and evaluate the overall ergonomic impact of the entire procedure. **Methods**: This single-center study involved 4 radiologists and focused on breast and abdominal ultrasonography. Kinematic data were recorded using the Noraxon Ultium Motion inertial system to track body movements during the ultrasound procedures. Five critical segments were identified while examining the liver, right kidney, left kidney, right breast, and left breast. Ergonomic assessment was performed using the Rapid Upper Limb Assessment (RULA) and Rapid Entire Body Assessment (REBA) methods, evaluating postural risks and physical strain during each segment and the whole procedure. **Results**: Both RULA and REBA assessments yielded median total scores of 6.0–7.0 and 6.0–7.5, respectively, reflecting consistently medium to high musculoskeletal loading. Examinations of the left breast and left kidney were associated with the most demanding postures. These elevated scores demonstrate that abdominal and breast ultrasonography imposes substantial ergonomic strain, potentially increasing the risk of work-related musculoskeletal disorders. **Conclusions**: The high ergonomic risk scores indicate an urgent need to modify scanning techniques and workstation design to reduce musculoskeletal strain in sonographers. Implementing ergonomic improvements is essential to prevent occupational injuries and promote long-term health.

## 1. Introduction

Ultrasonography is the most widespread diagnostic imaging modality. Ultrasound examinations are performed by physicians as well as representatives of other medical professions, both for diagnostic purposes and during interventional procedures. The versatility and safety of ultrasonography contribute to the growing number of ultrasound examinations performed worldwide [1]. This may cause work overload and the development of work-related musculoskeletal diseases (WMSDs) in sonographers. According to our previous study, 65.6% of sonographers experience pain during or shortly after performing ultrasound examinations, most commonly reported in the cervical and lumbosacral spine, shoulder and wrist [2,3,4,5,6,7]. Discomfort caused by poor working conditions may affect the course and accuracy of the examination as well as its result. To counteract WMSDs, associations related to healthcare and medical ultrasonography have proposed standards and recommendations regarding work ergonomics for ultrasound specialists [8,9,10,11]. These preventive principles are mostly based on observational studies of forced body positions and subjective opinions regarding discomfort accompanying sonographic examinations and incorporate recommendations developed for similar workstations, including computer work [12,13]. To date, there are only a few studies that have quantitatively measured joint loads and even fewer that have used dedicated equipment for measurements of the body kinematics in sonographers [14,15,16,17,18]. Various validated measurement methods are used to identify incorrect postures and points of physical overload as well as to assess the risk of WMSDs, including the Rapid Upper Limb Assessment (RULA), the Rapid Entire Body Assessment (REBA), the Ovako Working Posture Analysis System (OWAS) or the National Institute for Occupational Safety and Health (NIOSH) regulations [19,20,21,22,23]. Both RULA and REBA methods implement a point converter for the position angles of the arm, forearm and wrist, as well as the neck, trunk and legs. Measurements are taken in the sagittal plane separately for each of the upper limbs. The final score includes additional points for coronal parameters such as raised shoulder, abducted arm, and moving the forearm and the wrist sideways from the midline, making RULA/REBA methods highly suitable for analyzing sonographers’ body position.

The purpose of this study was to measure, analyze and quantify the typical musculoskeletal loads in sonographers performing breast and abdominal ultrasound examinations with a dedicated biomechanic methodology. Identifying the most burdensome positions will allow for the development of bioengineering solutions and workstation improvements. Subjective findings such as greater trunk rotation and reaching demand led to the hypothesis that left-sided examinations are associated with higher ergonomic risk.

## 2. Materials and Methods

### 2.1. Participants

This investigative, single-center study initially included 5 radiologists, three women and two men aged 29 to 39, who were substantially trained in breast and abdominal ultrasonography. The experience of the study participants ranged from 12 months (in this case, if at least 20 ultrasound examinations were performed per week) to 12 years.

The exclusion criteria for study participants, apart from the lack of required experience, included:

- Previous musculoskeletal surgeries;
- History of serious injuries;
- The presence of endoprostheses or skeletal stabilizing implants;
- Chronic and symptomatic diseases of the musculoskeletal system;
- Acute pain or exacerbation of chronic pain during the study period;
- Chronic or occasional (within 7 days preceding the study) use of painkillers, anti-inflammatory drugs, and antiepileptic drugs.

Ultrasound examinations were performed during routine patient follow-up in the Department of Diagnostic Imaging and Interventional Radiology of the University Clinical Center, Katowice, Poland. Sonographers performed examinations in the same setting, using the same equipment, and were sitting on a stool without lumbar support (indicated as the most common type of seat in our previous survey). Patients were lying on a typical, narrow examination table (52 cm high, 56 cm wide) with an adjustable headrest. All participants were right-handed with body characteristics within the range of normal anthropometric values defined as a body mass index (BMI) of 18.5–24.9: ID1—168 cm and 62 kg (BMI 21.97); ID2—167 cm and 60 kg (BMI 21.51); ID3—176 cm and 68 kg (BMI 21.95); and ID4 (male)—173 cm and 73 kg (BMI 24.39). Each participant performed complete breast and abdominal ultrasound twice, amounting to 10 sets of breast and abdominal ultrasound examinations. A single set consisted of abdominal and breast examinations performed on one patient in continuity. A total of 10 patients were involved.

Both doctors and patients gave informed written consent to participate in the study. The study protocol has been approved by the bioethics committee of the Medical University of Silesia in Katowice, Poland (BNW/NWN/0052/KBI/2/24).

As a result of the data quality verification process, all incomplete recordings and those containing any registration errors, due to incorrect data transfer caused by the sensor’s signal interference, were excluded. Ultimately, 4 out of the 10 recorded sessions from 4 radiologists (ID1–ID4), i.e., three women and one man, qualified for the analysis.

### 2.2. Kinematic Analysis

**Figure 1:**
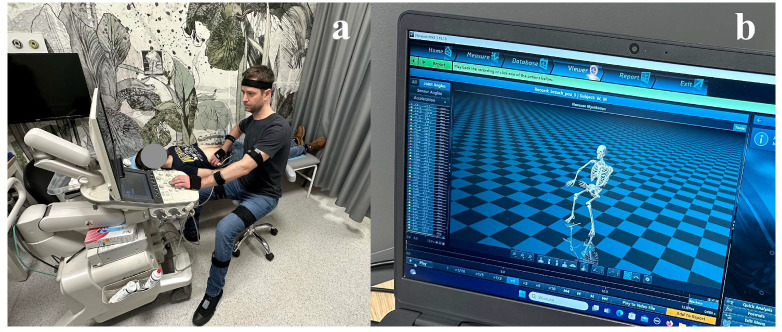
Kinematic evaluation during the testing phase: (**a**) motion sensors attached to the sonographer’s body and (**b**) software visualization of motion analysis in real time.

Sonographer kinematics were monitored using the Noraxon Ultium Motion system (Noraxon USA Inc., Scottsdale, AZ, USA). Sixteen sensors were attached to the participants according to the previously described protocol (Figure 1) [24]. The sensors were synchronized with the Ultium system via its proprietary radio transmission, ensuring real-time coordination of the data streams. Data were sampled at 200 Hz, with sensors calibrated in a standardized standing pose (feet hip-width apart, knees and elbows fully extended, arms resting at the sides, and rigid body position maintained throughout the calibration procedure). Sensor data was recorded in real time and analyzed with Noraxon MR3.18 software.

**Figure 2:**
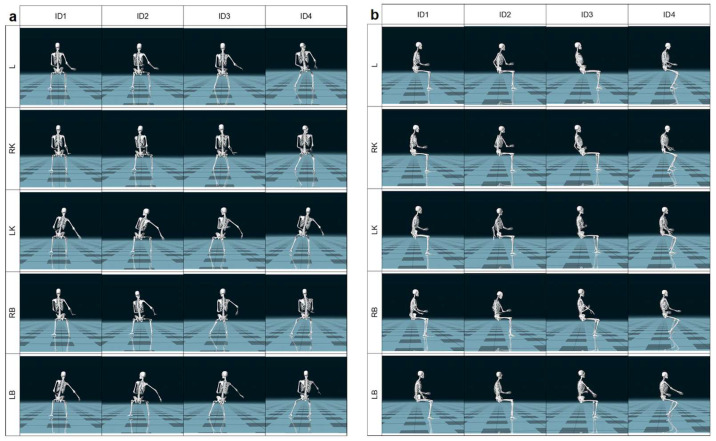
Identification of 5 critical physicians’ body positions in coronal (**a**) and sagittal plane (**b**). ID1–ID4—sonographer identification; L—liver; LB—left breast; LK—left kidney; RB—right breast; RK—right kidney.

Based on the analysis of the kinematic recordings, five critical positions of the transducer were identified, located over the right breast (RB), left breast (LB), liver (L), right kidney (RK) and left kidney (LK) (Figure 2). These examination segments were determined objectively by detecting the most demanding body positions that lasted at least 2 s, including trunk tilt and limb flexion, as well as based on the subjective reports of discomfort. The body position during visualization of the pancreas was insignificantly different to that of L but caused less subjective discomfort. The same observation was made with the imaging of RK and the spleen. Therefore the decision was made to focus on organs leading to more severe complaints in the specific examined region. The analysis included shoulder joint abduction, elbow joint flexion, dorsal/palmar wrist flexion, head flexion in the sagittal plane, and trunk flexion in the sagittal plane. Hip and knee joint flexions were additionally considered for the REBA method.

### 2.3. Biomechanical Analysis

The angle values measured by the Noraxon software MR3.18 algorithm were obtained automatically from the Inertial Measurement Unit (IMU) sensors (Noraxon USA Inc., Scottsdale, AZ, USA) and calculated according to the RULA and REBA methods using the official assessment sheets. A comparison of the results allowed a more detailed identification of ergonomic risk factors [19,20]. To perform the analysis, data recorded with the Noraxon Ultium Motion system were segmented according to a previously described framework. This approach allowed for the identification of anatomical structures most burdened within the musculoskeletal system. Additionally, complete recordings of abdominal and breast examinations were analyzed to assess the overall ergonomic risk and the potential for occupational diseases resulting from tasks performed by sonographers. The ergonomic assessment in both methods is conducted using images or video recordings captured in the sagittal plane [25,26,27].

#### 2.3.1. RULA Method

The first step in the RULA method involves evaluating flexion at the shoulder joint, followed by flexion at the elbow joint, wrist dorsiflexion/palmar flexion, and wrist twist. Based on these measurements, Group Score A is determined, which pertains to the upper body. Two additional indicators are then incorporated: Muscle Use Score (non-static posture—frequent changes in position more than 4 per minute) and Force/Load Score (less than 4.4 lb—holding only the ultrasound transducer). Next, Group B is assessed—this includes the posture of the head, trunk, and legs. As the clinician was seated without a backrest and the legs were in contact with the ground, the legs were considered as providing support. Similarly to Group A, indicators of Muscle Use Score (non-static posture) and Force/Load Score (again below 4.4 lb) were added.

The ergonomic risk levels, as defined by the RULA method, are defined as follows: 1–2—acceptable posture; 3–4—low risk, further investigate, change may be needed; 5–6—medium risk, further investigate, change soon; and ≥7—high risk, investigate and implement change.

#### 2.3.2. REBA Method

In the REBA method, Group A refers to the posture of the head, trunk, and legs. The legs were assigned the maximum score, as the physician was in a seated position. Subsequently, the Force/Load Score was assessed—here, the lowest value (<11 lb) was assigned, as the physician was only holding the ultrasound probe. Next, Group B was evaluated, corresponding to the upper extremities. An additional component, the Hand Coupling Score, was also assessed. In this case, a value corresponding to Well-Fitting Handle and Mid-Range Power Grip was selected, as the ultrasound probe was ergonomically shaped and fit well in the hand. In the final stage of the analysis, the Activity Score was determined, with a point added for repeated small-range actions (more than 4 per minute).

The levels of ergonomic risk according to the REBA method are defined as follows: 1—negligible risk; 2–3—low risk, change may be needed; 4–7—medium risk, further investigate, change soon; 8–10—high risk, investigate and implement change; and ≥11—very high risk, implement change.

### 2.4. Statistical Analysis

The RULA and REBA scores were analyzed using MATLAB R2023a (MathWorks, Natick, MA, USA). For each specified anatomical location (L, RK, LK, RB, and LB) and group (Group A, Group B, and Total Score) the median and interquartile range (IQR; 25th–75th percentile) were calculated using the MATLAB functions median() and prctile(). The results are presented in the standard format: median (Q1–Q3).

In order to perform the inference-based comparison, the RULA/REBA results were assessed using Friedman and pairwise Wilcoxon signed-rank tests. Statistical significance was set at a *p*-value below 0.05.

## 3. Results

The evaluation was conducted using two ergonomic assessment methods, namely RULA and REBA. The results are presented separately according to the method used.

### 3.1. RULA Analysis

**Figure 3:**
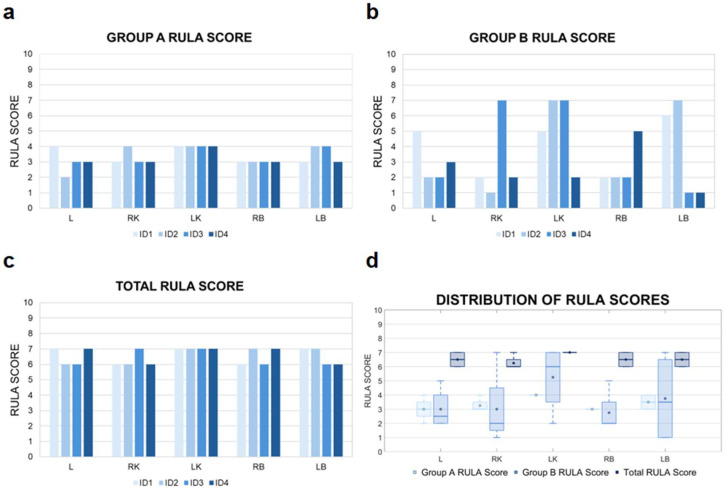
RULA score results. Detailed data for Group A are shown in (**a**), while data for Group B are presented in (**b**). The total RULA scores, reflecting whole-body ergonomic assessment, are illustrated in (**c**). Distribution of RULA values corresponding to the evaluation of specific anatomical locations are presented in (**d**). ID1–ID4—sonographer identification; L—liver; LB—left breast; LK—left kidney; RB—right breast; RK—right kidney.

The results obtained using the RULA method are presented collectively in Figure 3.

In the analysis conducted for Group A, imaging of the LK was consistently associated with the highest level of musculoskeletal load. It is important to emphasize that due to the fixed position of the ultrasound machine and the operator’s placement on the right side of the patient, accessing the left side of the body necessitated a non-physiological working posture. This included twisting and leaning the torso to the left, along with increased shoulder flexion of the right upper limb.

For anatomical structures located closer to the examiner’s working position—such as the RK or RB—a more neutral and ergonomically favorable posture could be maintained. The individual RULA scores for Group A indicate that LK imaging generates loading levels within the range of 3–4. Other anatomical structures received scores in the 2–3 range.

In Group B the highest levels of individual musculoskeletal load occurred during the imaging of the LK and LB, which were scored the highest by most examiners (up to 7 RULA points). These high scores primarily resulted from torso positioning—significant forward flexion, lateral bending, and rotation. Thus, torso posture was the main factor influencing the final score for this group. An exception was ID4, for whom the highest load was recorded during RB imaging due to individual differences in scanning technique—unlike the other participants, whose torso was positioned approximately parallel to the subject (the physician’s pelvis was perpendicular to the subject’s pelvis), ID4’s torso was turned more away from the subject (Figure 2a). Combined Total RULA scores indicated a high overall musculoskeletal load, with medians ranging from 6.5 to 7.0 points across all structures.

The analysis of median RULA scores for Group A indicated that the highest upper body load occurred during imaging of LK and LB due to the need for trunk rotation and leaning toward the examined region. Interquartile ranges were relatively narrow for most structures, indicating low variability between diagnosticians and consistent musculoskeletal strain patterns (Table 1). For Group B, the highest median RULA score loads were observed for LK (6.0; 3.5–7.0) and LB (3.5; 1.0–6.5). This suggests that imaging structures located further from the operator require more forced postures, increasing lower body strain and resulting in greater inter-individual variability.

The analysis of RULA scores for diagnosticians’ posture throughout the entire duration of the examination revealed that although the average values in Groups A and B may appear relatively low, the overall median total score was 6.5 (Table 2).

#### RULA Inference-Based Comparison

In Group A, representing the lower body, the Friedman test did not reveal statistically significant differences between anatomical sites (*p* = 0.764, Kendall’s W = 0.115). Similarly, in Group B, representing the upper body, no statistically significant differences were observed (*p* = 0.474, Kendall’s W = 0.220). Most pairwise comparisons within both groups using the Wilcoxon signed-rank test were also not significant (*p* > 0.05), although several effect sizes (r) were moderate to large, suggesting meaningful practical differences between specific sites. In the combined analysis (Total RULA Score), which includes both upper and lower body data, the Friedman test did not indicate a statistically significant difference (*p* = 0.406, Kendall’s W = 0.25), reflecting relatively low variation across anatomical sites. Although pairwise Wilcoxon comparisons in the total sample did not reach statistical significance (*p* > 0.05), the effect sizes (r) showed moderate practical differences between certain anatomical regions, highlighting areas of relatively higher ergonomic load. These findings suggest that, while statistical significance was limited within individual postures, the combined analysis reveals clear practical differences in RULA scores across anatomical sites. The effect sizes emphasize the presence of meaningful biomechanical variations, pointing to specific regions with higher ergonomic risk (Table 3 and Table 4).

### 3.2. REBA Analysis

**Figure 4:**
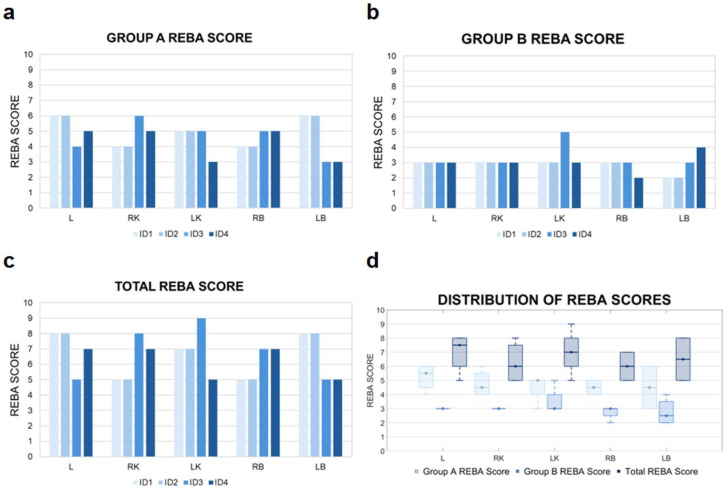
REBA score results. A detailed analysis of the outcomes for Group A is shown in (**a**), while the corresponding results for Group B are provided in (**b**). The total REBA scores, reflecting the ergonomic assessment of the whole body, are illustrated in (**c**). Meanwhile, the distribution of REBA scores assigned to each of the evaluated anatomical regions are visualized in (**d**). ID1–ID4—sonographer identification; L—liver; LB—left breast; LK—left kidney; RB—right breast; RK—right kidney.

The aggregated results obtained using the REBA method are presented in Figure 4.

The individual analysis of REBA scores for Group A revealed the highest levels of musculoskeletal strain during imaging of L and LB (range of 5–6). These medium-risk scores were primarily attributable to torso positioning—including significant forward flexion, rotation, and lateral bending—which contributed most heavily to the overall strain in this parameter. Torso posture proved to be the decisive factor influencing the REBA outcomes in this group.

The REBA score analysis for Group B indicates low ergonomic risk levels within the 2–3 point range. An exception was noted in the case of LK imaging by examiner ID3, where the REBA score reached 5, indicating a moderate risk level. This result highlights the necessity for a more detailed analysis and potential adjustments in workstation configuration or scanning technique. Other anatomical structures including L, RK, and both breasts showed no elevated scores, implying more ergonomic examiner postures during those procedures.

The analysis of total REBA scores, reflecting the overall musculoskeletal load, showed consistently elevated values across all examined cases. The highest loads were associated with imaging of the LK, with scores up to 9, indicating a high overload risk. Lower scores were observed during imaging of the RK and RB, denoting moderate strain that still warrants ergonomic attention.

The highest median REBA scores for Group A and relatively narrow IQRs were observed for L and LK, indicating consistently high upper body loads associated with these locations (Table 5). For Group B, REBA medians were lower for most structures (3.0–3.5), except for LK, which showed an IQR of 3.0–4.0, reflecting moderate variability between diagnosticians.

The analysis of REBA scores for examiner postures throughout the entire examination procedure reveals that, although the average values in Groups A and B fall within the low-to-moderate range, the overall mean score reaches 5 (Table 6).

#### REBA Inference-Based Comparison

In Group A (lower body), the Friedman test did not reveal statistically significant differences between anatomical sites (*p* = 0.764, Kendall’s W = 0.115). Similarly, in Group B (upper body), no statistically significant differences were observed (*p* = 0.474, Kendall’s W = 0.220). Most pairwise comparisons within both groups using the Wilcoxon signed-rank test were also non-significant (*p* > 0.05). Nevertheless, several effect sizes (r) ranged from moderate to large, suggesting meaningful practical differences between anatomical sites despite the lack of statistical significance. In the combined analysis (Total REBA Score), which included both upper and lower body data, the Friedman test indicated a statistically significant difference (*p* = 0.040, Kendall’s W = 0.625), reflecting substantial overall variation across sites. Although pairwise Wilcoxon comparisons in the overall sample did not reach statistical significance (*p* > 0.05), the corresponding effect sizes were consistently large (|r| ≈ 0.73–1.83), indicating pronounced practical differences between specific anatomical sites. These findings suggest that, while statistical significance was limited—likely due to the small sample size—the effect sizes reveal clear and meaningful patterns of biomechanical load. The results highlight which body regions consistently experience higher REBA scores, underscoring the practical relevance of these differences even in the absence of formal significance (Table 7 and Table 8).

## 4. Discussion

In the analysis conducted for RULA Group A, relating to the upper limb, the examination of the LK was associated with the highest level of musculoskeletal load, which included twisting and leaning the torso to the left, along with increased shoulder flexion. LK scans also showed the highest loads when analyzing mean RULA values. Other organs received scores in the 2–3 range, suggesting postures that are close to acceptable or require only moderate attention. This classification highlights the need to pay particular attention to the ergonomics of scanning structures located contralaterally to the dominant hand to prevent upper body musculoskeletal overload. The REBA score analysis for Group B indicated moderate ergonomic risk levels, suggesting a need only for ongoing monitoring or minor adjustments. Generally low-to-moderate scores may reflect favorable working conditions for the upper limb. However, even isolated instances of elevated strain emphasize the importance of accounting for individual postural differences and scanning techniques in ergonomic risk evaluation. Regardless of the specific anatomical location, total REBA scoring confirmed a significant and persistent risk of musculoskeletal strain, highlighting the necessity for ergonomic optimization in clinical environments. The particularly high values observed during imaging of left-sided structures likely stem from the need to operate in torso rotation and forward bending.

In RULA Group B, which concerns the loads occurring in the torso, the highest levels of musculoskeletal loads occurred during the imaging of the LK and LB. Based on the average RULA scores for the anatomical structures in both groups, it can be concluded that the total musculoskeletal load is high. For almost all evaluated structures—except RK—the total RULA score was 7, indicating a need for immediate intervention. The individual analysis of REBA scores for Group A revealed the highest levels of musculoskeletal strain during imaging of the LB and L. In the case of both RULA and REBA body trunk analyses, the high scores were primarily attributable to significant forward flexion, rotation, and lateral bending of the torso. Due to the proximity of the right side of the patient to the examiner, a more ergonomic posture was often possible during the imaging of right-sided structures. These cases were associated with lower REBA scores, reflecting improved torso positioning and reduced overall musculoskeletal burden. Although no instances of very high risk levels were observed in this group, the findings clearly underscore the importance of ergonomic consideration—particularly with respect to torso alignment—when planning workstation setups and examination techniques to minimize musculoskeletal overload.

Consistently high total RULA scores (>5) in this sample indicate musculoskeletal strain across all anatomical regions, particularly the LK, L, and both breasts. High and comparable RULA scores for nearly all structures suggest that the positioning of sonographers and scanning techniques generate significant muscular tension and postural overload across various body regions, regardless of the specific area being examined. Comparable results were seen in the case of total REBA scores, which showed consistently elevated values across all examined cases ranging from 5 to 9. REBA scores point to a high ergonomic risk particularly during imaging of the LB and L, where scores reached 8.

When evaluating load according to examination-specific regions of interest, the most demanding structures could be identified. For breast ultrasound, the highest medians and ranges were observed for LB. For abdominal ultrasound, LK was the most physically demanding, with ranges reflecting high variability depending on individual technique. Findings indicate that locations opposite the operator’s dominant hand generate the greatest musculoskeletal strain and therefore support the study hypothesis.

Across both RULA and REBA assessments, posture-specific analyses for Group A (lower body) and Group B (upper body) did not reveal statistically significant differences between anatomical sites, as indicated by Friedman tests (*p* = 0.360, W = 0.272 for RULA Group A; *p* = 0.764, W = 0.115 for REBA Group A; *p* = 0.087, W = 0.507 for RULA Group B; and *p* = 0.474, W = 0.220 for REBA Group B). Pairwise Wilcoxon signed-rank tests also did not reach significance (*p* > 0.05) within individual postures. Despite the absence of statistical significance, effect sizes (r) were moderate to large in several comparisons, indicating meaningful practical differences between specific anatomical regions. In the combined analysis (Total), which included both upper and lower body data, the Friedman test for RULA did not achieve significance (*p* = 0.406, W = 0.25), whereas the Friedman test for REBA indicated a statistically significant difference (*p* = 0.040, W = 0.625), reflecting greater overall variation across anatomical sites in the latter assessment. Wilcoxon pairwise comparisons in the total samples remained non-significant for both tools; however, effect sizes consistently highlighted practical differences, particularly in the L, LK, and RB, which emerged as the most ergonomically stressed regions. These findings suggest that, although formal statistical significance was limited—likely due to the small sample size—the magnitude and direction of effect sizes reveal clear patterns of biomechanical load. Both RULA and REBA converge in identifying the same high-risk anatomical sites, with REBA providing slightly greater sensitivity in detecting total-body ergonomic variation. Overall, these results emphasize the practical importance of addressing L, LK, and RB in ergonomic interventions to mitigate biomechanical overload and potential musculoskeletal risk.

Previous research has shown similar conclusions regarding RULA scores. One study examined the musculoskeletal loads according to the RULA method during ultrasound of the thyroid gland, abdominal cavity and venous thrombosis of the lower limbs [14]. The measurements were performed based on the analysis of the video recording during the examinations and using electrogoniometers (Biometrics Ltd., Ladysmith, VA, USA). In this study, all scans showed significant Group A scores resulting from awkward upper limb postures, forceful manual exertions, and applied static muscle contraction. Group B scores were consistently found to be higher for left-sided organs, which required the most immediate attention. In a study by Habes et al,. the main ergonomic stress factors observed during abdominal and transvaginal examinations were right shoulder flexion and abduction, sustained static forces, and various types of transducer grips [15]. Friesen et al. determined detailed values of sonographers’ joint angles while preforming various ultrasound procedures in which trunk-side flexion toward the patient ranged from 5–10°, shoulder abduction from 10–60°, shoulder flexion from 30–40°, and elbow flexion from 30–110° [16]. A different video-based study showed that sonographers spend 68% of scanning time with >30° shoulder abduction, 63% of time with >30° shoulder outward rotation, and 37% of time with the neck bent forward, laterally or twisted > 20° [17]. The shoulder was unsupported or static for 73% of scanning time mainly during carotid ultrasound compared with abdominal, obstetrical or lower limb examinations. For larger patients more wrist flexion and extension were used to reach the closer and further areas of the abdomen [15]. Sonographers also had to twist the neck to the left to view the monitor, flex and abduct the left shoulder, and extend the elbow to operate the control panel. Based on the EMG analysis of the shoulder and back muscles, the static amplitude probability distribution functions exceeded 3% maximum velocity contraction, corresponding to a medium risk rating for shoulder and neck disorders [17]. The same study reported an average probe grip force during scanning of 4 kg with the peak forces reaching over 27 kg [28]. The minimal applied force of 1 kg provides little or no opportunity to rest and is associated with increased risk of hand/wrist cumulative trauma disorders, including carpal tunnel syndrome. Additional identified factors contributing to the loads experienced by the sonographers were bed width (higher loads observed for wider beds) and the lack of elbow support [15].

The analysis of scores for sonographers’ postures throughout the entire duration of both breast and abdominal examinations revealed that the average values in Groups A and B were low and moderate for RULA and REBA methods, respectively. Nevertheless, the overall mean total score reached 7 for RULA, indicating a high level of musculoskeletal strain, and 5 in case of REBA, corresponding to a notable level of strain. The persistence of such high values underscores the urgent need to optimize working conditions and adapt scanning techniques to effectively reduce musculoskeletal loading among sonographers.

Continuous monitoring and the implementation of ergonomic interventions are essential for improving workplace comfort and occupational safety. The main emphasis is on maintaining a neutral body position during ultrasound examinations, education and training in ergonomics as well as sensible management of the work schedule [9]. Additional emphasis is placed on equipment and its ergonomic configuration during diagnostic sonography [29]. Before beginning the examination, it is recommended to adjust the control panel and monitor as well as the chair and examining bed, utilize a footrest and seats with lumbar support. Additional devices include cushions, ergonomic probes, cable braces and forearm/arm/shoulder support systems. The few available reports on prototypes of assistive devices leave considerable room for further biomechanical investigations [30]. A promising perspective already being dynamically explored is robot-assisted ultrasound (RAUS), which has the potential to facilitate future ultrasound diagnostics while relieving sonographers of their workload and minimizing the risk of WMSDs [31].

### 4.1. Study Limitations

One limitation of our study is the small sample size. Only 4 out of 10 sets of examinations from 4 out of 5 sonographers were analyzed due to the presence of incomplete data or incorrect data transfer caused by the sensor’s signal interference. The inclusion criterion for the analysis was a complete recording of both breast and abdominal ultrasound, enabling the selection of key moments of the examination for each critical position.

Another limitation of the study that limits its generalizability is the lack of assessment of the influence of sonographers’ and patients’ anthropometric data on kinematic results. However, a reliable assessment would require examining a large group of individuals with a wide range of body types.

A possible combined effect of body position and exercised pressure level generated by the sonographer over the patient’s body was not taken into consideration in this study and should be elucidated in future research. Additionally, the force/load in the central portions of the upper and lower abdomen, in order to assess the aorta, pancreas, renal arteries and urinary bladder, were not evaluated.

### 4.2. Study Strengths

To the best of our knowledge, this is the first study utilizing motion sensors to assess the kinematics of sonographers. The use of sensors allowed us to obtain the most appropriate and precise measurements and therefore to exclude any measurement errors related to the assessment of body position in a single plane. It is also the first study to analyze biomechanics and postural loads during breast ultrasound examinations.

## 5. Conclusions

The analyses indicate that LB is the most demanding structure for breast ultrasound, whereas the LK poses the highest load during abdominal ultrasound. For both RULA and REBA methods, the total scores for LK and LB ranged from 6.5–7.0 and 8.0–9.0, respectively. The overall median IQR for both methods reached 7.0 for LK and 6.5 for LB. Findings indicate that locations opposite the operator’s dominant hand generate the greatest musculoskeletal strain and therefore support the study hypothesis. Anatomical structures located closer to the examiner’s working position determined a more neutral and ergonomically favorable posture. Adopting a forced body position during ultrasound examinations seems unavoidable; therefore solutions should be sought that will reduce physical strain, including training in work ergonomics and developing supporting devices that relieve the musculoskeletal system.

## Figures and Tables

**Table 1:** Median RULA scores calculated for specific anatomical locations.

	Rula Score		
	Group A	Group B	Total
	MEDIAN (Q1–Q3)	MEDIAN (Q1–Q3)	MEDIAN (Q1–Q3)
L	3.0 (2.5–3.5)	2.5 (2.0–4.0)	6.5 (6.0–7.0)
RK	3.0 (3.0–3.5)	2.0 (1.5–4.5)	6.0 (6.0–6.5)
LK	4.0 (4.0–4.0)	6.0 (3.5–7.0)	7.0 (7.0–7.0)
RB	3.0 (3.0–3.0)	2.0 (2.0–3.5)	6.5 (6.0–7.0)
LB	3.5 (3.0–4.0)	3.5 (1.0–6.5)	6.5 (6.0–7.0)

L—liver; LB—left breast; LK—left kidney; RB—right breast; RK—right kidney.

**Table 2:** RULA scores calculated for the entire duration of both breast and abdominal examinations.

	ID1	ID2	ID3	ID4	Median (Q1–Q3)
Group A RULA Score	3	3	2	4	3.0 (2.5–3.5)
Group B RULA Score	2	2	5	2	2.0 (2–3.5)
Total RULA Score	6	6	7	7	6.5 (6–7)

ID1–ID4—sonographer identification. Ergonomic risk: 1–2—acceptable posture; 3–4—further investigation, change may be needed; 5–6—further investigation, change soon; ≥7—investigate and implement change.

**Table 3:** Friedman test results for Posture A, Posture B, and Total RULA scores.

Group	*p*-Value	W	Significant
Posture A	0.360	0.272	No
Posture B	0.087	0.507	No
Total RULA Score	0.406	0.250	No

W—Kendall’s coefficient of concordance.

**Table 4:** Pairwise Wilcoxon signed-rank test results for RULA scores.

Group	Pair	*p*-Value	r	Significant
Group A	L vs. RK	0.75	−1.278	No
	L vs. LK	0.50	−1.826	No
	L vs. RB	1.00	−1.278	No
	L vs. LB	0.75	−1.278	No
	RK vs. LK	1.00	−1.095	No
	RK vs. RB	0.50	−0.730	No
	RK vs. LB	1.00	−1.278	No
	LK vs. RB	0.25	0.365	No
	LK vs. LB	1.00	−1.461	No
	RB vs. LB	0.50	−1.826	No
Group B	L vs. RK	0.75	0.365	No
	L vs. LK	0.50	−1.826	No
	L vs. RB	1.00	−1.461	No
	L vs. LB	0.125	−1.826	No
	RK vs. LK	0.625	−0.730	No
	RK vs. RB	0.50	−1.826	No
	RK vs. LB	0.25	−1.826	No
	LK vs. RB	0.75	0.548	No
	LK vs. LB	0.25	−1.826	No
	RB vs. LB	0.25	−1.826	No
Total RULA Score	L vs. RK	1.00	−1.278	No
	L vs. LK	1.00	−1.278	No
	L vs. RB	1.00	−0.365	No
	L vs. LB	0.50	−1.826	No
	RK vs. LK	1.00	−1.278	No
	RK vs. RB	1.00	−0.365	No
	RK vs. LB	0.50	−1.826	No
	LK vs. RB	1.00	−0.365	No
	LK vs. LB	0.50	−1.826	No
	RB vs. LB	0.25	−1.826	No

r—effect size; L—liver; LB—left breast; LK—left kidney; RB—right breast; RK—right kidney.

**Table 5:** Median REBA scores calculated for specific anatomical locations.

	Reba Score		
	Group A	Group B	Total
	MEDIAN (Q1–Q3)	MEDIAN (Q1–Q3)	MEDIAN (Q1–Q3)
L	5.5 (4.5–6.0)	3.0 (3.0–3.0)	7.5 (6.0–8.0)
RK	4.5 (4.0–5.5)	3.0 (3.0–3.0)	6.0 (5.0–7.5)
LK	5.0 (4.0–5.0)	3.0 (3.0–4.0)	7.0 (6.0–8.0)
RB	4.5 (4.0–5.0)	3.0 (2.5–3.0)	6.0 (5.0–7.0)
LB	4.5 (3.0–6.0)	2.5 (2.0–3.5)	6.5 (5.0–8.0)

L—liver; LB—left breast; LK—left kidney; RB—right breast; RK—right kidney.

**Table 6:** REBA scores calculated for the entire duration of both breast and abdominal examinations.

	ID1	ID2	ID3	ID4	Median (Q1–Q3)
Group A REBA Score	4	4	3	4	4.0 (3.5–4.0)
Group B REBA Score	3	3	1	3	3.0 (2.0–3.0)
Total REBA Score	5	5	5	5	5.0 (5.0–5.0)

ID1–ID4—sonographer identification. Ergonomic risk: 1—negligible risk; 2–3—low risk; 4–7—medium risk.

**Table 7:** Friedman test results for Posture A, Posture B, and Total REBA scores.

Group	*p*-Value	W	Significant
Group A	0.764	0.115	No
Group B	0.474	0.220	No
Total REBA Score	0.040	0.625	Yes

W—Kendall’s coefficient of concordance.

**Table 8:** Pairwise Wilcoxon signed-rank test results for REBA scores.

Group	Pair	*p*-Value	r	Significant
Group A	L vs. RK	1.000	−0.365	No
	L vs. LK	0.500	1.095	No
	L vs. RB	0.500	0.000	No
	L vs. LB	1.000	−1.461	No
	RK vs. LK	1.000	0.365	No
	RK vs. RB	1.000	−1.461	No
	RK vs. LB	1.000	−0.730	No
	LK vs. RB	1.000	−0.730	No
	LK vs. LB	0.750	−0.548	No
	RB vs. LB	1.000	−1.095	No
Group B	L vs. RK	0.250	1.461	No
	L vs. LK	0.250	0.365	No
	L vs. RB	0.500	0.000	No
	L vs. LB	1.000	−1.095	No
	RK vs. LK	0.750	−0.548	No
	RK vs. RB	1.000	−1.826	No
	RK vs. LB	0.500	−1.826	No
	LK vs. RB	1.000	−0.730	No
	LK vs. LB	0.750	−0.548	No
	RB vs. LB	1.000	−1.095	No
Total REBA Score	L vs. RK	1.000	−1.826	No
	L vs. LK	0.500	−1.826	No
	L vs. RB	1.000	−1.826	No
	L vs. LB	0.500	−0.730	No
	RK vs. LK	0.500	−1.826	No
	RK vs. RB	1.000	−1.826	No
	RK vs. LB	0.500	−0.730	No
	LK vs. RB	0.500	−0.730	No
	LK vs. LB	0.125	1.826	No
	RB vs. LB	0.500	−0.730	No

r—effect size; L—liver; LB—left breast; LK—left kidney; RB—right breast; RK—right kidney.

## Data Availability

The original contributions presented in this study are included in the article. Further inquiries can be directed to the corresponding author.
